# Unilateral Giant Myelolipoma of the Adrenal Gland: A Case Report

**DOI:** 10.7759/cureus.77510

**Published:** 2025-01-15

**Authors:** Huber Díaz, Ricardo A Millán Flores, Héctor A Ancona Pérez, Brenda V Ventura Trujeque

**Affiliations:** 1 Urology, Mexican Institute of Social Security Hospital Regional No. 1, Mérida, MEX; 2 Urology, Faculty of Medicine, Autonomous University of Yucatan, Mérida, MEX

**Keywords:** adrenal glands, adrenal myelolipoma, adrenal surgery, benign neoplasms, laparoscopic treatment

## Abstract

Myelolipoma of the adrenal gland is a rare, benign, non-functioning tumor characterized by the presence of adipose tissue and bone marrow elements. We present the case of a 48-year-old woman with intermittent left flank pain and an incidental finding of an adrenal tumor on computed tomography. The patient underwent laparoscopic tumor resection due to the large size of the tumor. The decision to perform surgery was based on the tumor size and the patient’s symptoms. Laparoscopic adrenalectomy is a safe surgical technique with low complication rates and shorter hospital stays.

## Introduction

Adrenal myelolipomas are rare, benign, and non-functional neoplasms composed of mature adipose tissue and hematopoietic progenitor cells, originating in the adrenal cortex. They were first described in 1905 by Gierke and owe their name to Oberling [1]. The size is variable and generally small (<6 cm), although giant tumors have been reported that can cause symptoms such as abdominal pain, hematuria, and high blood pressure [2].

While most myelolipomas are smaller than 5 cm, some have been described as giant, reaching up to 34 cm in size and weighing almost 6 kg. The therapeutic approach is expectant for small and asymptomatic tumors, while surgery is recommended for symptomatic cases or tumors >6 cm due to the risk of spontaneous rupture with retroperitoneal hemorrhage, which can be fatal [3].

The incidence of these tumors ranges from 0.08% to 0.4%. They are often discovered incidentally during autopsies, surgeries, or imaging studies (ultrasound or computed tomography) conducted for unrelated reasons. Myelolipomas constitute 15% of adrenal incidentalomas [2]. They frequently occur between the fifth and seventh decades of life, without sex predominance. The adrenal location of myelolipomas is the most common. Although large tumors are not usually symptomatic, surgical resection is recommended for lesions larger than 4-5 cm due to the risk of hemorrhage [3]. We present a case of a rare symptomatic variant of adrenal myelolipoma in a 48-year-old woman and discuss the relevant literature.

## Case presentation

A 48-year-old female presented to our hospital with the following medical condition: she denied smoking and drug addiction. She had type 2 diabetes mellitus under treatment and a surgical history of abdominal hysterectomy and open cholecystectomy. The patient had been experiencing intermittent pain in the left flank for two months, which required the administration of analgesics. She denied hematuria and weight loss. On physical examination, the abdomen was distended due to adipose tissue, normal bowel sounds were auscultated, and abdominal masses were not palpable.

**Figure 1:**
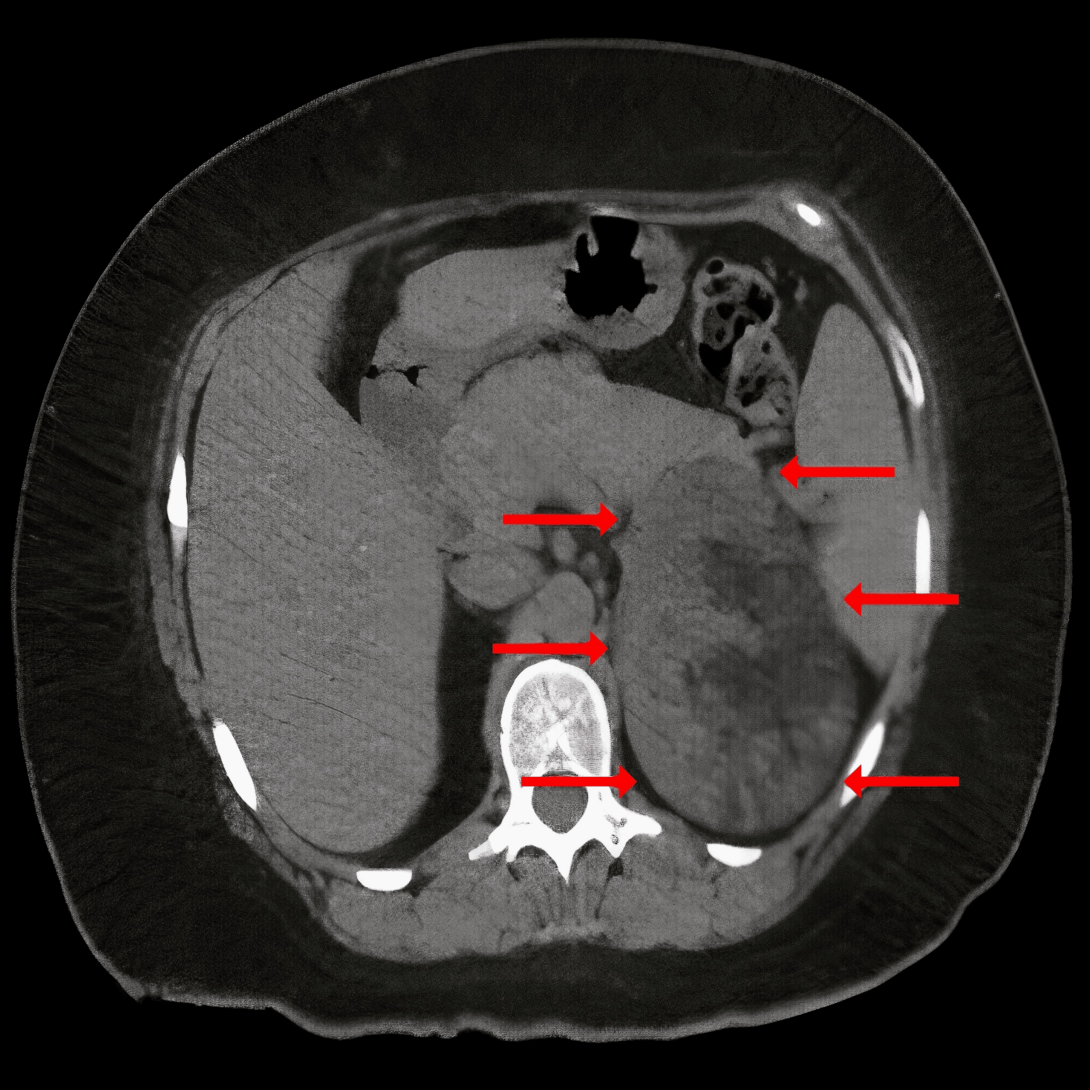
Computed axial tomography showing a mass located in the adrenal gland with heterogeneous density.

**Figure 2:**
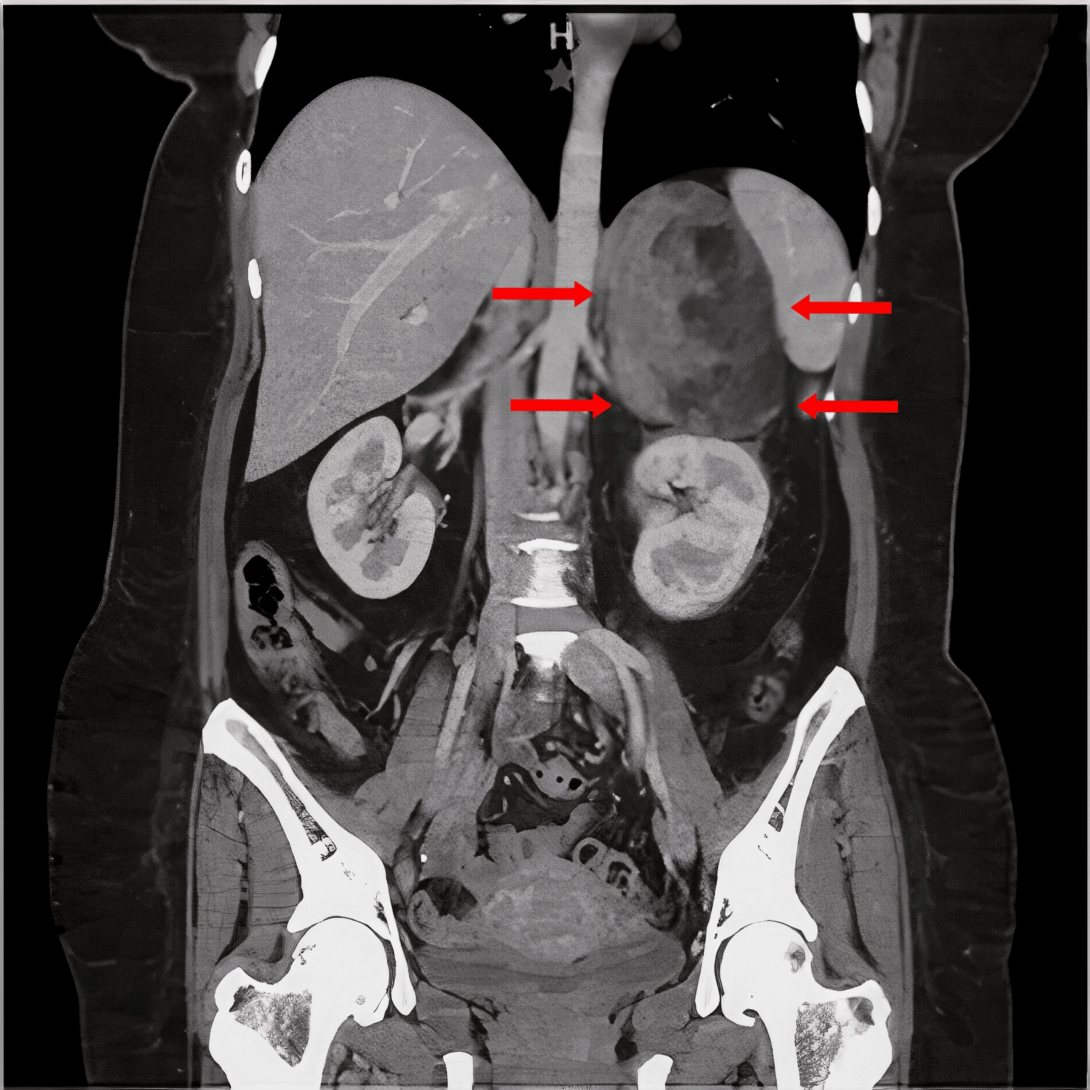
Computed tomography coronal section showing adrenal location mass with the dimensions of 90 x 69 x 62 mm that has contact with the spleen.

She underwent a computed tomography (CT) scan. CT features at the anatomical site of the left adrenal gland revealed a well-defined ovoid tumor with heterogeneous content, including hypodense areas (5 HU), fat, and calcification. The tumor measured 90 × 69 × 92 mm. Upon administration of a contrast medium, the hypodense parts showed enhancement of up to 44 HU (Figures 1, 2).

A left laparoscopic adrenalectomy was performed using a transperitoneal approach. Four trocars were placed: the first at the level of the 11th rib in the pararectal line, the second and third trocars using the triangulation technique, and the fourth trocar for the assistant 3 cm above the anterior superior iliac spine. Intraoperative findings revealed loose adhesions, a firm myelolipoma adhered to the spleen, one adrenal artery originating from the renal artery, and one vein. The artery and vein were clipped using Hem-o-lok. The operating time was 72 minutes, with a blood loss of 50 mL. A Penrose-type drain was left in place and removed the following day.

The surgery was completed without any intraoperative or postoperative complications. The patient was discharged on postoperative day 3, tolerating food, walking by the next day, having bowel movements, and showing postoperative laboratory results within normal parameters.

**Figure 3:**
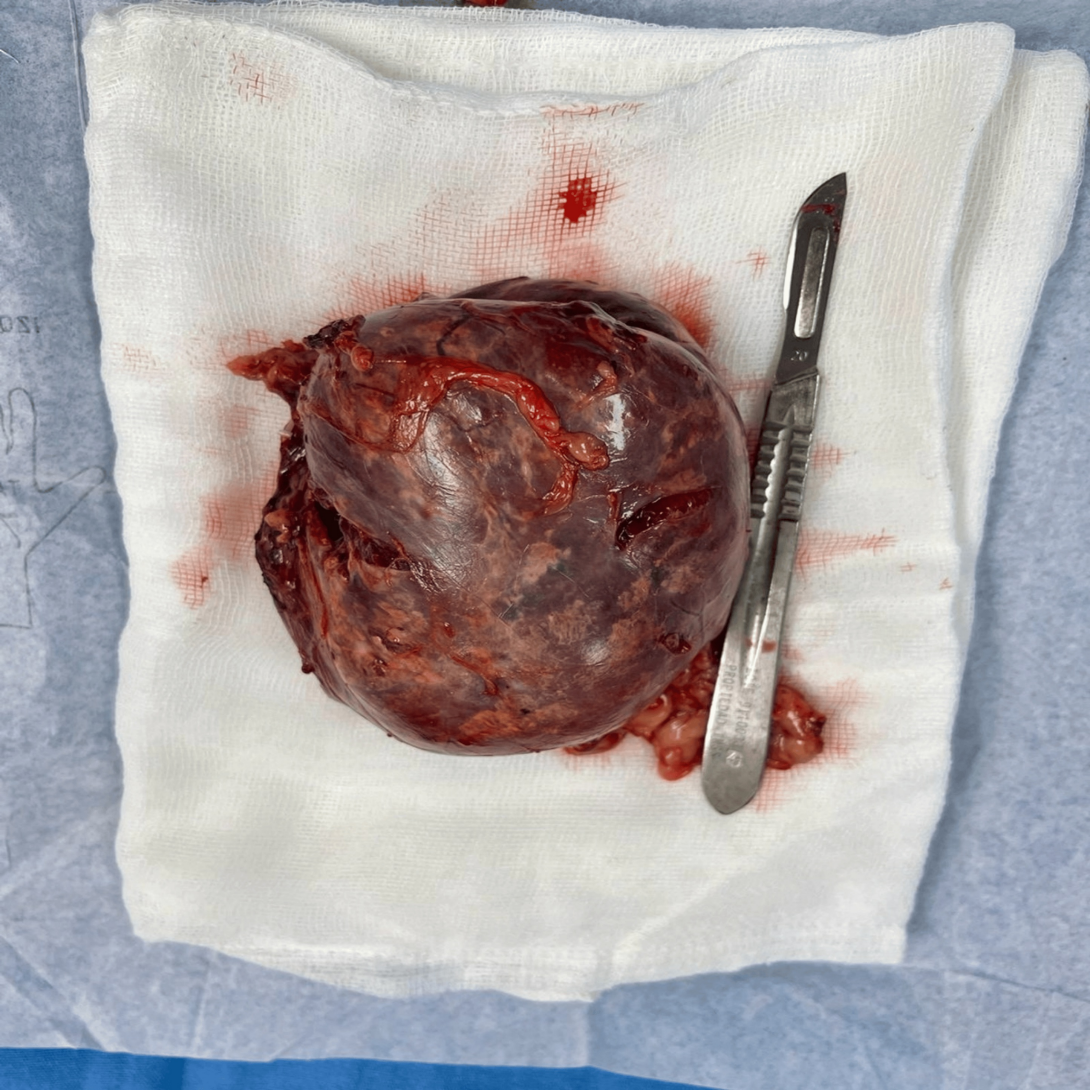
Gross features showing a tumor with lobulated surface partially covered by fat tissue measuring 12 × 7 × 6 cm.

Macroscopically, the tumor was soft, with a smooth yellow external surface and a solid yellow section alternating with areas of hemorrhagic appearance, measuring 120 × 70 × 60 mm (Figure 3). It was concluded that the myelolipoma had been completely resected.

**Figure 4:**
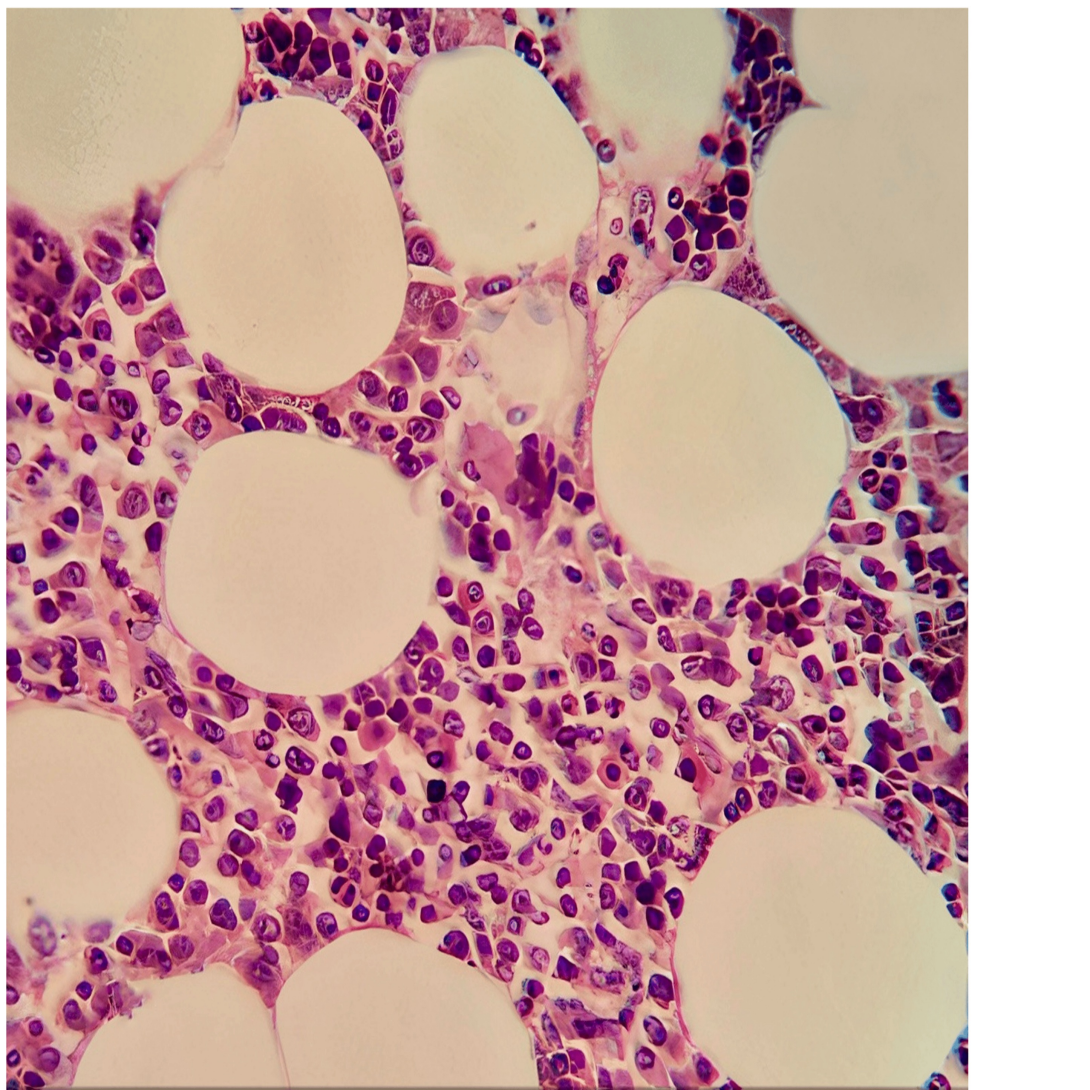
Microscopic view with hematoxylin-eosin stain at 100× magnification, showing hypercellularity corresponding to hematopoietic cells and adipocytes.

Histologically, it is a benign mesenchymal neoplasm with pushing edges, consisting primarily of mature adipose tissue and nests of trilinear extramedullary hematopoiesis [4]. No lipoblasts or atypical stromal cells were observed. Necrosis or calcification was not present (Figure 4).

## Discussion

The objective of this study is to demonstrate that myelolipomas larger than 7 cm can be managed by laparoscopic adrenalectomy, as there are currently no guidelines designating laparoscopic management of large lesions as the gold standard.

Historically, older literature recommends treating lesions larger than 7-8 cm with open surgery. However, laparoscopy now offers several advantages, including shorter hospital stays, less pain, and reduced bleeding [2].

Adrenal myelolipoma is a rare variant of adrenal lipoma. In our case, the patient was 48 years old, which falls within the mean age group, and the tumor size was 12 cm, significantly larger than the baseline of 4 cm [4]. Myelolipomas are usually asymptomatic but can cause flank pain and abdominal discomfort due to compression of surrounding structures, tumor necrosis, rupture, or bleeding, which can lead to hemorrhagic shock [5].

Due to the tumor size of 12 cm and the risk of local compression, our patient was counseled for surgical resection. Imaging studies are essential in the management of myelolipomas. CT is the most sensitive method, revealing hyperdense areas corresponding to myeloid tissue and hypodense areas corresponding to adipose tissue. On MRI, the tumor typically appears hyperintense on T1-weighted images and hypointense on T2-weighted images due to the predominance of adipose tissue [6]. Because imaging findings can overlap with those of other tumors with adipose components, differential diagnoses should include liposarcomas, pheochromocytomas (characterized by higher densities of adipose tissue >30 HU), and extramedullary hematopoiesis (EMH) [7].

Histopathological analysis revealed the presence of mature adipose tissue and hematopoietic elements in variable proportions, along with cells of the myeloid, lymphoid, or megakaryocytic series, as well as lymphoid aggregates and plasma cells. Areas of calcification and ossification are commonly identified [7]. The treatment of adrenal myelolipomas must be individualized. Small asymptomatic lesions, less than 3-4 cm in size, can be monitored for 1-2 years with CT or MRI, though some advocate for clinical follow-up without radiological studies [8]. Surgery is indicated for symptomatic lesions larger than 5 cm or when malignancy is suspected. Asymptomatic tumors that grow during follow-up should also be surgically removed. According to the literature, laparoscopy is the most commonly used surgical approach, with no size limit for its indication. However, this approach is not recommended in cases of adhesions or infiltration of neighboring organs [9].

Gagner performed the first laparoscopic adrenalectomy in 1992 using a transperitoneal approach to access the adrenal gland. Currently, laparoscopic access is one of the main indications for urologic surgery, eliminating the need for large lumbotomy incisions to treat adrenal lesions, which previously resulted in high morbidity and mortality rates of up to 40% [5]. The laparoscopic technique is now considered the reference standard for the treatment of benign, functional, or non-functional adrenal lesions smaller than 12 cm. Transperitoneal laparoscopic adrenalectomy can be performed using the supine or lateral anterior approach [8]. The supine approach allows for bilateral adrenalectomy without repositioning the patient, whereas the lateral approach provides more working space.

The patient was positioned in a 30° to 45° backward-tilted position. After abdominal insufflation, three or four trocars were placed along the right costal margin. The duodenum was dissected using the Kocher maneuver, and a trocar was used to retract the liver. The renal and adrenal veins were dissected. Surgical clips were applied: two on the vein side and one on the specimen side. A harmonic scalpel was effectively used for dissection. The specimen was placed in an endoscopic bag and removed [10].

## Conclusions

Myelolipoma is a rare urological entity that has been increasingly detected due to the widespread use of diagnostic imaging methods, which often reveal it incidentally. Laparoscopic adrenalectomy is a safe surgical technique with low complication rates and shorter hospital stays.

Laparoscopic resection of the tumor has shown favorable outcomes, even in cases involving large masses, as demonstrated in this article. If surgery is indicated for a benign adrenal mass, a minimally invasive approach is recommended. This aligns with the new 2023 guidelines by the European Society of Endocrinology.
